# Near-infrared spectroscopy: recent advances in infant speech perception and language acquisition research

**DOI:** 10.3389/fpsyg.2014.00916

**Published:** 2014-08-15

**Authors:** Judit Gervain

**Affiliations:** Laboratoire Psychologie de la Perception, CNRS - Universite Paris DescartesParis, France

**Keywords:** language learning, language developmental, speech perception, brain specialization for language, near-infrared spectroscopy, developmental cognitive neuroscience

Near-Infrared Spectroscopy (NIRS) is a relatively novel and increasingly popular optical imaging technique that has revolutionized brain research in the developmental populations (Villringer and Chance, 1997; Lloyd-Fox et al., 2009; Gervain et al., 2011). After more than a decade of technological development, NIRS has become a reliable, easy-to-use and efficient tool to explore the linguistic and cognitive abilities of neonates and young infants, opening new vistas for the investigation of language acquisition and cognitive development. This Research Topic covers the latest advances in these areas brought about by NIRS imaging. The main focus is to highlight innovative and foundational studies that go beyond methodological issues and advance our theoretical understanding of infant and child development. Contributions from the pioneers of this method are selected, illustrating how NIRS has allowed developmental researchers to ask theoretically relevant questions that more traditional methods couldn't address.

The first two contributions, by Fava et al. (2011) and Benavides-Varela et al. (2011), cover general theoretical issues and methodological principles. They provide a critical, but constructive overview of theoretical questions about linguistic and cognitive development that have been asked, outline challenges that the NIRS community still needs to face and offer recommendations for optimal experimental designs and data interpretations practices.

These general contributions are followed by a series of empirical papers exploring a key issue in the study of the neural correlates of language learning and development, the nature and origins of the brain specialization for speech and language. While it is well established that in the majority of right-handed adults, language is preferentially processed in the left hemisphere (e.g., Friederici, 2005), the reasons for and the ontogenetic origins of this left lateralization have so far been less well understood, partly because the field lacked a safe, fully non-invasive, participant-friendly brain imaging method with which to probe the infant brain. NIRS has filled this gap, opening up the way for exciting new discoveries about the brain specialization for speech and language in young babies (e.g., Pena et al., 2003; Sato et al., 2012). Five experimental articles in the current volume contribute to this exciting inquiry. May et al. (2011) compare newborn infants' brain responses to the native language, spoken by the mother during pregnancy, and to an unknown language, in an attempt to investigate how prenatal experience with speech might shape the brain specialization for language. Telkemeyer et al. (2011), Arimitsu et al. (2011) as well as Minagawa-Kawai et al. (2011) take a different approach, seeking to identify the acoustic, spectro-temporal properties of the speech signal might underlie brain specialization. In adults, it has been shown that fast-changing sounds or sounds modulated in time preferentially recruit areas in the left hemisphere that are part of the language network, while slowly changing sounds or sounds modulated spectrally tend to engage the right hemisphere (Zatorre et al., 2002; Hickok and Poeppel, 2007). This offers a potential explanation for why most language stimuli, with their fast phoneme and syllable transitions, activate the left hemisphere, with prosody being the only aspect of language that is processed in the right hemisphere. However, adults have extensive experience with language, leaving open the issue of causation. Telkemeyer et al. (2011), Arimitsu et al. (2011), and Minagawa-Kawai et al. (2011) now test these hypotheses on newborns and young infants using different temporally and spectrally modulated tone stimuli, asking whether the observed hemispheric specializations are the causes or the results of lateralized language processing. As an innovative extension of the research on early brain specialization for speech, Sato et al. (2011) investigate whether, and if yes, how this specialization might be different in an atypical population, stuttering children and adults.

The last three contributions inquire into more advanced or higher level mechanisms of language processing and comprehension. Homae et al. (2011) used a new method of NIRS data analysis to explore functional connectivity and networks in 3-month-old infants at rest and while they listen to speech stimuli, identifying a large-scale brain network engaged in language processing. Wagner et al. (2011) explore the neural correlates of learning abstract linguistic rules at 7 and at 9 months of life and show important developmental changes signaling infants increased specialization for and attunement to language structure. Tabea Brink et al. (2011) study the brain mechanisms underlying the understanding of empathy in verbal and picture-based stories in pre-school children, an age which is believed to be crucial for the development of emotional and cognitive empathy.

It is my hope that these NIRS studies further our understanding of language and cognitive development and bring us closer to bridging the gap between brain, mind and behavior at the very beginning of life.

## Conflict of interest statement

The author declares that the research was conducted in the absence of any commercial or financial relationships that could be construed as a potential conflict of interest.
